# Turning Mushy Lipids into Fruity Notes: Unlocking Lactone Biosynthesis Potential in Fat Industry Lipid Waste

**DOI:** 10.3390/foods14244326

**Published:** 2025-12-15

**Authors:** Jolanta Małajowicz, Katarzyna Wierzchowska, Karina Jasińska, Agata Fabiszewska

**Affiliations:** Department of Chemistry, Institute of Food Sciences, Warsaw University of Life Sciences-SGGW, 159C Nowoursynowska Street, 02-776 Warsaw, Poland

**Keywords:** valorization, oily waste, biotransformations, lactones, *Yarrowia lipolytica*

## Abstract

Waste from the fat-processing industry represents a challenging stream due to its physicochemical properties and environmental impact. Valorization through recovery and reuse offers ecological, economic, and social benefits. This study focused on mushy lipid residues generated during cold pressing of oilseeds (sunflower, flax, blue poppy, hemp, black cumin, and walnut) and evaluated their potential for lactone biosynthesis. The waste was analyzed for protein and fat content, while fatty acid profile, acid and peroxide values, oxidation stability, and health-related indices characterized the extracted oils. Polyphenol content and antioxidant activity of the residues were also determined. Subsequently, the waste was used as a substrate in biotransformation processes with *Lactiplantibacillus plantarum* and *Yarrowia lipolytica*. The results showed high protein (13.1–19.4%) and fat levels (65.0–77.3%) across all residues. The lipid fractions were rich in monounsaturated and polyunsaturated fatty acids, comprising nearly 90% of the total fatty acids, with oleic and linoleic acids being the dominant components. These features highlight their strong valorization potential, particularly for the microbial synthesis of aroma-active lactones. Under the applied conditions, the production of γ-dodecalactone and δ-decalactone reached 0.76 g/L and 1.62 g/L, respectively, confirming the suitability of cold-press residues as substrates for sustainable biotechnological applications.

## 1. Introduction

The global production of cold-pressed vegetable oils generates increasing amounts of viscous residues rich in lipids and bioactive compounds, which remain largely unexploited despite their potential for valorization. In response to regulatory pressure and circular-economy goals, the sector increasingly focuses on valorizing these by-products [1].

Valorization transforms waste into value-added products through material recycling, such as recovery of proteins, fats, fiber, and bioactive compounds for feed, nutraceuticals, cosmetics, or food additives; and energy recovery (biogas, bioethanol, biodiesel) or biological processes, including bioconversion, composting, and fermentation [2]. Carbohydrate-rich substrates, such as whey, molasses, and lignocellulosic waste, support large-scale production of single-cell protein and bioethanol fermentation [3,4]. Distillery residues, though high in organic matter, provide polysaccharides, polyphenols, and short-chain fatty acids [5]. Fruit-processing by-products are rich in antioxidants, including anthocyanins, flavones, flavonols, and stilbenes [6,7]. Raspberry pomace extract increased polyphenols in fruit purées threefold [8], while berry pomace flour improved fermented drinks [4,9]. Apple pomace enriched animal feeds and reduced antibiotic use; drying stabilized it and enhanced bakery quality [10,11].

In oil processing, by-products include oil cakes and meals formed after pressing or defatting of the oil. Edible cakes from flax, peanut, sunflower, and rapeseed contain 15–50% protein, lipids and minerals, and are used in ruminant and aquaculture feeds [12,13]. Waste cooking oils (WCO) are increasingly utilized as bio-based modifiers and rejuvenators in asphalt binders, improving flexibility and fatigue resistance [14,15]. Furthermore, lipid-rich wastes, including fats, oils, and grease (FOG), are valuable co-substrates for anaerobic digestion, enhancing methane yields by 30–50% [16,17]. Lipid waste also supports cost-efficient biosurfactant production, as demonstrated for rhamnolipids from olive oil effluent and sophorolipids from deodorization condensates [18,19].

There are many examples of biotechnological valorization of lipid food waste, including *Yarrowia lipolytica*, a yeast that converts WCO and lipid-rich effluents into citric acid and high-value lipids. *Cutaneotrichosporon curvatus* and *Cryptococcus* spp. utilize glycerol and fatty acid residues from biodiesel waste to produce single-cell oils and microbial biomass; meanwhile, *Aspergillus niger* transforms agro-industrial lipid by-products into citric acid [20,21,22]. Oleaginous microorganisms can accumulate more than 20% of their dry mass as triacylglycerols under nitrogen limitation; they stand as a potential target group of cells aimed for biotechnological conversion of lipid-rich waste materials [23,24]. The yeast *Y. lipolytica*, which has a GRAS status, grows on hydrophobic wastes and exhibits strong lipase activity [25].

Valorization of fatty acid industry waste, which poses a significant environmental burden, is a key aspect of biotechnological processes for producing high-value-added products. Implementing circular economy principles for lipid waste can provide dual benefits, reducing technological consequences and environmental impacts while simultaneously increasing economic efficiency. These aspects prompted us to research the use of waste in the synthesis of fragrance compounds. The fragrance market is constantly evolving, and efficient biotechnologies based on cheap, waste substrates are gaining popularity. The high percentage of the lipid fraction in waste, dominated by unsaturated fatty acids (MUFAs) and PUFAs, makes their valorization path, based on biotransformations resulting in cyclic esters—lactones—an attractive option.

Lactones, cyclic esters found in microorganisms, plants, and insects, exhibit characteristic aromas and diverse biological activities. γ- and δ-lactones impart peach and dairy flavors, with γ-decalactone being particularly important for the food and fragrance industries [26,27]. Biotechnological methods for the synthesis of lactones are primarily based on the beta-oxidation of hydroxylated fatty acids, which occur in the peroxisomes of microorganisms. An example is the biosynthesis of gamma-decalactone from the precursor ricinoleic acid (the main component of castor oil obtained from the *Ricinus communis* plant [26]). This is the best-known method of gamma-decalactone biosynthesis so far. However, due to the limited amount of naturally occurring hydroxy acids and the high cost of obtaining the precursor, which is comparable to the product yield, alternative methods for lactone synthesis are being sought.

Considering the concept of sustainable development and the current trend toward a circular economy, biotransformation processes utilizing unsaturated acids in the synthesis of lactones appear promising. Lactone biosynthesis from unsaturated fatty acids proceeds via three main steps: (i) hydrolysis of triacylglycerols to free fatty acids; (ii) enzymatic hydration of double bonds mediated by bacterial hydratases (e.g., oleate or linoleate hydratase) [28,29]; and (iii) β-oxidation of intermediate hydroxy acids by fungal or yeast enzymes, followed by spontaneous lactonization under acidic conditions [30].

This lactone synthesis pathway requires the use of mixed cultures in biotransformation processes—bacteria are responsible for the hydration of unsaturated bonds, and yeast for the beta oxidation cycle. In the biosynthesis of lactones, the decision was made to use whole microbial cells instead of commercial enzyme systems due to their economic and practical advantages. Whole-cell catalysts are less expensive, characterized by higher stability and greater metabolic flexibility, which is essential both at the research and industrial stages.

The ability of microorganisms to hydrate unsaturated fatty acids was first discovered in 1960 by Wallen et al. [31], who obtained 10-hydroxystearic acid through the bacterium *Pseudomonas species*. Since then, many more microorganisms, mainly, have been described to catalyze this reaction [32,33,34,35,36,37].

In this work, we undertook research on the synthesis of lactones using mushy waste lipid materials from the fat industry. To our knowledge, there are no studies that exploit the potential of mushy lipid residues in the synthesis of fragrance compounds, and no comparative studies have taken into account such a wide range of analyzed parameters of oilseed residues in the context of waste management. Therefore, this study aimed to evaluate the valorization potential of viscous residues generated during cold-press oil from black cumin, sunflower, poppy, hemp, flax, and walnut seeds by using them as substrates for microbial biosynthesis of lactone aroma compounds through biotransformation. The study analyzed the concentration of ô-decalactone and γ-dodecalactone, which resulted from the biotransformation pathways of linoleic acid and oleic acid, respectively, present in the waste. The research was enriched by a detailed analysis of mushy lipid residues, making it a comprehensive project in terms of their biotechnological valorization.

## 2. Methods

### 2.1. Materials

The research material was a mushy, oily waste product resulting from the cold-pressing of oil from oilseed plants (Table 1). Six unctuous, oily substances were examined, constituting waste from raw materials such as walnut, sunflower, hemp seed, flax, black cumin, and blue poppy seed. The material came from Oleovital Company (Grajów, Poland). The mushy lipid waste was delivered immediately after pressing of oil and stored refrigerated at 4 °C. Analyses were performed within 2 weeks of obtaining the research material.

*Yarrowia lipolytica* KKP379 was obtained from the Collection of Industrial Microorganisms at the Prof. Wacław Dąbrowski Institute of Agricultural and Food Biotechnology (State Research Institute, Warsaw, Poland). The strain *Lactiplantibacillus plantarum* came from the collection of microbial cultures at the Warsaw University of Life Sciences (Warsaw, Poland). The microorganisms were stored in a −18 °C freezer in 20% (*v*/*v*) glycerol until used.

Ingredients for microbiological media, including peptone, yeast extract, glucose, and MRS medium, were purchased from BTL (Łódź, Poland). Inorganic reagents, including KOH, H_2_SO_4_, K_2_SO_4_, CuSO_4_, KI, Na_2_CO_3_, and organic solvents, including hexane, toluene, dichloromethane, chloroform, acetic acid, ethanol, and methanol, were purchased from Avantor Performance Materials S.A. (Gliwice, Poland). Folin–Ciocalteu reagent, gallic acid, and DPPH radical reagent were from Sigma Aldrich (Saint Louis, MO, USA).

### 2.2. Analytical Methods

#### 2.2.1. Dry Mater Content

To determine the dry matter content, approximately 20 g of each mushy, oil waste was weighed into previously weighed falcons and dried to constant weight in a KBC-65G dryer at 100 °C for approximately 24 h. After drying and cooling in a desiccator, the samples were reweighed, and the percentage of dry matter was calculated from the difference in weight.

#### 2.2.2. Protein Determination by Kjeldahl Method

Protein was determined using a Tecator Digestion System (Hilleroed, Denmark) and a Tecator Distillation Unit, Kjeltec 1026. Approximately 1.0 g of sample was weighed into a digestion tube, and 7.0 g of catalyst (prepared by mixing K_2_SO_4_ and CuSO_4_·5H_2_O) and 12 mL of concentrated H_2_SO_4_ were added. The tubes with samples were introduced into the digester. The digestion temperature was 420 °C, and the duration was one hour. After digestion, the released ammonia was distilled from the cooled solution into a solution of saturated boric acid with the addition of a mixed indicator (methyl red + bromocresol green). The ammonia solution in boric acid was determined by titration with a standard 0.01 M hydrochloric acid solution (until the initial color was obtained, the same as before the absorption of ammonia).

Protein content is calculated from Equations (1) and (2), respectively:

Kjeldahl nitrogen (%) = ((V_S_ − V_B_) × M × 14.01)/(W × 10), (1)

Crude protein (%) = % Kjeldahl N × F(2)

where

V_S_ = volume (mL) of standardized acid used to titrate a test;
V_B_ = volume (mL) of standardized acid used to titrate reagent blank;
M = molarity of standard HCl;
14.01 = atomic weight of N;
W = weight of the sample analyzed;
10 = factor to convert mg/g to percent
F = factor to convert N to protein—5.3.

#### 2.2.3. Fat Content Determination

Approximately 20 g of each of the tested oily wastes was weighed into Falcone centrifuge tubes, and 40 mL of dichloromethane was added. A 10 min extraction was performed using a rotator with a shaker function Bionovo (Emeryville, CA, USA), 85 rpm/min), followed by centrifugation in a Centrifuge MPW-352 (Warsaw, Poland) centrifuge for 10 min at 8000 rpm. The extraction was repeated three times. After evaporation of the solvent, the percentage of fat in each oily waste was calculated from the difference in mass between the empty flask and the flask with fat.

##### Characteristics of the Fat Fraction in Oily Waste

1. Acid value determination by potentiometric titration

0.1–0.2 g of previously extracted fat was weighed. Then, 70 mL of a 1:1 (*v*/*v*) mixture of toluene and ethanol was added to each sample. Titration was performed using a 0.1 molar KOH solution in a Titralab (Hach Lange Sp. z o.o., Wroclaw, Poland) automatic analyzer. The results of this determination were expressed in mg KOH/g fat.

2. Peroxide value determination

Peroxide value was determined by titration. A sample of 0.5–0.6 g was weighed, then 30 mL of a 2:3 (*v*/*v*) mixture of chloroform and acetic acid was added to each sample, followed by 1 mL of a saturated potassium iodide (KI) solution. The prepared samples were thoroughly mixed using a magnetic stirrer and left in a dark cabinet for 5 min. After this time, the solutions were titrated with sodium thiosulfate using a Titralab (Hach Lange Sp. z o.o., Wroclaw, Poland) automatic analyzer.

3. Determination of the oxidative induction time

Pressure Differential Scanning Calorimetry (PDSC) was used to define the oxidative stability of oil from oil waste. To determine the induction time for the oxidation reaction of oil, experiments were carried out with the help of a DSC Q20 apparatus (TA Instruments, New Castle, DE, USA) linked to a high-pressure chamber. 3–4 mg of fat was weighed into aluminum vessels designed for DSC analysis. Oil samples were place in the oxygen atmosphere and under a pressure of 1350–1400 kPa. Measurements were taken isothermally at 120 °C. Oxidation induction time was determined from PDSC curves. The result of the PDSC measurement was expressed in minutes.

4. Fatty acids composition analysis

The analysis of the fatty acid composition was carried out using the gas chromatography method. A GC YL6100 chromatograph (Young Lin Bldg., Anyang, Republic of Korea) was used with a flame ionization detector (FID) and a BPX70 capillary column with an internal diameter of 0.25 mm × length of 60 m and a film thickness of 0.25 μm. For analysis, fatty acids were converted into volatile derivatives by esterification with methanol, per the PN-EN ISO 5509:2001 standard [38]. The fatty acid methyl esters separation was performed under the following conditions: The initial temperature was 70 °C, and held for 0.5 min. Then, the temperature was increased at a rate of 15 °C/min to 160 °C, and then increased at a rate of 1.1 °C per minute to 200 °C, and further at a rate of 30 °C per minute to reach 225 °C. At this temperature, a hold was performed for 15 min. The injector and detector temperatures were 225 °C and 250 °C, respectively. Nitrogen was used as the carrier gas. Fatty acids were identified based on retention time compared with standards.

5. Determination of the lipid indices

Based on the profile of FAs, atherogenic index (AI), thrombogenic index (TI), and the value of hypocholesterolemic/hypercholesterolemic ratio (h/H) were calculated using the modified Formulas (3)–(5) proposed by Ulbright and Southgate [39] and Mierlită [40]:

AI = (C12:0 + (4 × C14:0) + C16:0)/(ΣPUFA)(3)

TI = (C14:0 + C16:0 + C18:0)/(0.5 × ΣMUFA + (0.5 × Σ*n*-6PUFA) + (3 × Σ*n* − 3PUFA) + (*n* − 3)/(*n* − 6) (4)

h/H = (ΣMUFA + ΣPUFA)/(C12 + C14:0 + C16:0)(5)

6. Determination of total polyphenols content

The total polyphenol content in oil samples extracted from oil waste was determined using the Folin–Ciocalteu method, with gallic acid (3,5,7-trihydroxybenzoic acid) as the reference standard. Five grams of oil were weighed into falcons, 30 mL of methanol was added, and the mixture was extracted for 10 min. It was then centrifuged in a Centrifuge MPW-352 for 10 min at 8000 rpm. For spectrophotometric determination, 0.2 mL of the clear fraction was collected. Then, 4.90 mL of water and 0.3 mL of Folin–Ciocâlteu reagent were added, followed by a 3 min wait. Subsequently, 0.6 mL of sodium carbonate was added. The prepared samples were kept in the dark for one hour and then measured spectrophotometrically at a wavelength of λ = 750 nm. Based on the previously prepared standard curve, the total phenolic content in the extracted oils was expressed as mg GAE (gallic acid equivalents) per g of oil.

7. Determination of antioxidant activity

The antioxidant activity was determined using the DPPH method, which involves the colorimetric measurement of the degree of reduction in DPPH free radicals (2,2-diphenyl-1-picrylhydrazyl). Absorbance was measured at a wavelength of 517 nm using methanol as the reference solution.

For analysis, 1 g of oil was weighed into a Falcon tube, and 5 mL of hexane and 5 mL of methanol were added. The samples were extracted and then centrifuged. After phase separation, 0.050 mL of the lower phase was removed, and a solution of the DPPH radical in methanol (2.95 mL) was added. The samples were mixed, allowed to stand in the dark for 20 min, and then the absorbance was measured. The results were expressed as the percentage of DPPH inhibition according to the formula:

[%] = A_b_ − A_s_/A_b_ × 100%,

A_b_—absorbance of the blank
A_s_—absorbance of the test sample.

#### 2.2.4. Chromatographic Analysis of Volatile Biosynthesis Products

The separation and identification of extracted components of the volatile fraction were performed in a gas chromatograph coupled to a GC-MS-QP2020 NX mass spectrometer from Shimadzu (Kyoto, Japan) using the HS-SPME-GCMS technique. The gas chromatograph was equipped with a ZB-5ms capillary column (length: 30.0 m, internal diameter: 0.25 mm, film thickness: 0.25 μm). Volatile compounds were extracted using a headspace microextraction technique (three-phase fiber, stationary phase: DVB/CAR/PDMS—Divinylbenzene/Carboxene/Polydimethylsiloxane, Supelco, Phenomenex). The injector temperature was 230 °C. A 1:30 flow splitter was used. The carrier gas (helium) flow rate in the column was 1.50 mL/min. The gas chromatograph oven temperature was maintained at 45 °C for 5 min. The temperature was then increased at a rate of 5 °C/min to 195 °C, followed by a rate of 25 °C/min to 270 °C, which was maintained for 5 min. The mass spectrometer was operated in TIC mode with a mass range of 40–400 Da. The junction temperature was 200 °C. The ion source temperature was 200 °C.

Identification of volatile compounds was performed by comparing the retention indices of the compounds with standards and databases and mass spectra from the NIST 11 and NIST 11s libraries. Sample preparation for analysis involved collecting 4 mL of the sample into a 20 mL chromatography vial. The vial was tightly closed with an aluminum cap. The fiber was conditioned for 30 min at 270 °C in the injector port. The sample was held at 45 °C for 20 min, and then volatile compounds were extracted using SPME at the same temperature for an additional 20 min. Desorption from the fiber took place in the injector port at 230 °C for 5 min. Each sample was analyzed in three replicates.

#### 2.2.5. Microbiological Culture with Added Mushy, Lipid Waste

##### Multiplication of *L. plantarum* and *Y. lipolytica* Cells

*L. plantarum* cells were grown in liquid MRS medium in Schott bottles. Bacterial culture was maintained in an incubator at 37 °C for 48 h.

*Y. lipolytica* yeast cells were grown in standard YPG medium, consisting of yeast extract (10 g/L), peptone (20 g/L), and glucose (20 g/L). The inoculum was prepared in a 250 mL flat-bottomed flask with a working volume of 50 mL. Incubation was carried out at 28 °C for 24 h, shaking at 140 rpm.

##### Microbiological Cultivation with the Addition of Lipid Waste, Targeting Lactone Biosynthesis

Ten grams of each waste product were weighed into 500 mL flat-bottom flasks, and 100 mL of MRS medium was added. The flasks were then autoclaved at 121 °C for 15 min. After the medium cooled, 0.5 g of lipase (*Aspergillus oryzae*, Sigma-Aldrich (Saint Louis, MO, USA), activity ≥ 20,000 U/g) was added, and the mixture was shaken for 6 h. After this time, the media were inoculated with lactic acid bacteria cells (inoculum concentration approx. OD = 0.5) and then incubated for 48 h at 37 °C. After a two-day incubation, the media were inoculated with yeast inoculum (inoculum concentration approx. OD = 0.25), and cultivation continued by incubating the flasks at 28 °C for 96 h, with shaking at 140 rpm. The control medium was prepared similarly, omitting the addition of oily waste.

After six days of biotransformation, volatile compounds were extracted from the medium and analyzed chromatographically to identify and quantify the lactones. For extraction, 2 mL samples were taken and acidified to approximately pH 3 with 0.1 M HCl, and then extracted with 2 mL of ethyl acetate. The internal standard was gamma-undecalactone (C_11_). The organic phase was dried over anhydrous magnesium sulfate, filtered, and then derivatized for GC-MS analysis.

#### 2.2.6. Statistical Analysis

The mean values and standard deviations were calculated using Microsoft Excel 2019. Statistical analysis was performed using Statistica 13 software (StatSoft Sp z o.o., Warsaw, Poland) with one-way analysis of variance (ANOVA) and Tukey’s test. Significant differences between samples were determined at *p* ≤ 0.05.

## 3. Results and Discussion

### 3.1. Physicochemical Characterization of Cold-Press Oil Residues

The residues obtained after cold pressing of oil-bearing seeds (walnut—WA, sunflower—SF, hemp—HS, flaxseed—FX, black cumin—BC, and blue poppy—PS) were analyzed for their physicochemical characteristics (Table 2). The dry matter content ranged from 5.50% (SF) to 25.00% (FX), while protein content varied between 13.11% (BC) and 19.44% (HS). The fat content was generally high (≈70% or above), except for BC (49.06%). Acid values (AV) ranged from 20.89 mg KOH/g oil (HS) to approximately 81 mg KOH/g oil (WA and BC), and peroxide values (PV) from 8.76 meq O_2_/kg (PS) to 52.16 meq O_2_/kg (FX). Total phenolic content (TPC) was low overall (0.04–0.11 mg GAE/g oil), with the highest values observed for SF and PS residues. DPPH inhibition values ranged from 9.00 to 33.00%, with the highest radical scavenging activity observed for sample BC, whereas HS showed the weakest response.

These results demonstrated substantial variability among the residues. The high lipid content confirmed that cold-press residues still retained significant amounts of oil, consistent with reports that oilseed cakes contained approximately 1–31% residual lipids and 14–52% protein, depending on the seed type. Such variability arose mainly from differences in oilseed species, pressing conditions, and extraction efficiency, which influenced both the residual oil and protein contents of the obtained cakes [41]. Residues with elevated acid values and peroxide values, such as WA, BC, and FX, likely underwent more extensive lipid hydrolysis and oxidation, leading to the formation of free fatty acids and peroxides that might have inhibited microbial activity. Similar trends were described for cold-pressed oils, where oxidation parameters varied widely depending on seed composition. For instance, Symoniuk et al. [42] reported peroxide values ranging from 0.95 meq O_2_/kg for linseed oil to as high as 81.93 meq O_2_/kg for black cumin oil. Ancuţa and Amariei [13] also noted substantial variability in the oxidative stability of oilseed cakes, associated with differences in fatty-acid profiles and residual oil content. These observations confirmed that lipid degradation depended strongly on both the degree of unsaturation and antioxidant retention, explaining the wide variation observed among the studied residues.

The relatively higher TPC in SF and PS residues suggested a greater presence of antioxidant compounds, which might have been beneficial for microbial transformation processes. Previous reviews on oilseed cakes have identified phenolic compounds as major bioactive antioxidants, thereby supporting the valorization of these residues within circular economy and biorefinery-oriented frameworks [13,43].

Overall, the sunflower and blue poppy residues appeared to be the most promising substrates for microbial biosynthesis of lactone aroma compounds, as they combined high fat content with moderate oxidation and the highest phenolic content. In contrast, walnut and black cumin residues, characterized by high AVs, required pre-treatment (water removal, filtration/clarification) or the use of more tolerant microbial strains. The flaxseed residue also showed potential, but needed stabilization due to its high peroxide value. These findings aligned with recent literature, which emphasized that the chemical quality of cold-press residues, particularly their lipid integrity and phenolic composition, played a crucial role in their valorization potential [44].

### 3.2. Fatty Acid Composition and Oxidative Stability of Residual Oils

The fatty acid profiles of the cold-press oil residues obtained from walnut (WA), sunflower (SF), hemp (HS), flaxseed (FX), black cumin (BC), and blue poppy (PS) are presented in Table 3. Polyunsaturated fatty acids (PUFAs) predominated in all residues, accounting for 53.46–76.45% of total fatty acids, followed by monounsaturated (MUFAs, 14.08–31.15%) and saturated fatty acids (SFAs, 5.97–15.39%). Linoleic acid (C18:2 *n*-6c) was the dominant component, ranging from 34.73% (BC) to 73.20% (PS). α-Linolenic acid (C18:3 *n*-3) was found in high amounts in FX (22.88%) and BC (18.02%), while it was absent in SF. Oleic acid (C18:1 *n*-9) ranged from 14.08 to 21.42%, and palmitic acid (C16:0) was the main SFA (up to 9.74% in PS). Minor fatty acids, such as arachidic (C20:0), eicosenoic (C20:1 *n*-9), and erucic (C22:1 *n*-9), were primarily found in BC, indicating a more diverse lipid profile.

Overall, the residues were rich in unsaturated fatty acids (MUFA + PUFA > 80%), confirming that they retain the essential lipid composition of their respective seeds even after cold pressing. PUFA-dominant profiles are reported for linseed and hemp cold-pressed cakes (with total unsaturated fatty acids typically exceeding 80%), whereas sunflower cake shows a substantially lower unsaturated fraction (~45%), reflecting strong inter-seed variability [41,44]. In our study, high PUFA levels in FX and PS (65–76%) indicate nutritionally valuable but lipids susceptible to oxidation, whereas BC—with the highest MUFA (31.15%) and SFA (15.39%) contents—may exhibit greater oxidative stability [45].

The predominance of linoleic and α-linolenic acids is particularly relevant from a biotechnological perspective, as these unsaturated fatty acids can serve as efficient precursors for microbial biosynthesis of γ- and δ-lactones via β- and ω-oxidation pathways. Previous reviews have highlighted the high content of C18:2 *n*-6 and C18:3 *n*-3 in flaxseed, hemp, and sunflower oilseed cakes, underscoring their potential as substrates for biotransformation into aroma-active compounds [43].

The oxidative stability of lipid fractions derived from cold-pressed oilseed residues was assessed using isothermal differential scanning calorimetry (DSC) under high-pressure oxygen. The obtained oxidation induction time (OIT) values are presented in Table 4. Significant differences were observed among the tested residues, with OIT values ranging from 3.12 min for sunflower (SF) to 39.00 min for hemp (HS). Intermediate oxidative stability was recorded for flaxseed (FX, 22.34 min) and black cumin (BC, 33.83 min), whereas walnut (WA, 10.49 min) and blue poppy (PS, 12.15 min) showed relatively short induction periods.

These results highlight the influence of fatty-acid composition and antioxidant retention on lipid oxidation resistance. The longest OIT values observed for HS and BC may be explained by their favorable fatty-acid characteristics and the possible presence of lipid–soluble antioxidants such as tocopherols, which delay oxidation initiation. Comparable findings were reported by Islam et al. [46], who observed OIT values of 42–58 min at 120 °C for cold-pressed hempseed oil, associated with high levels of γ- and α-tocopherols (up to 74 mg/100 g). Similarly, Tura et al. [47] demonstrated that hempseed oil with higher antioxidant retention exhibited slower peroxide formation during accelerated storage. The study by Siol et al. [48] regarding hemp oil (HO) and pumpkin seed oil (PO) blends measured oxidative stability using PDSC analysis at 120 °C. Pure hemp oil was characterized by the shortest maximum oxidation time, measuring 30.36 min, due to its high content of polyunsaturated fatty acids (PUFA). The addition of the more stable pumpkin oil (PO) extended this time, and the value showed a strong positive correlation with monounsaturated (MUFA) and saturated fatty acid (SFA) content (r = 0.86), and a strong negative correlation with PUFA (r = −0.86).

In contrast, SF and WA residues exhibited the shortest OIT values, consistent with their high linoleic acid (C18:2 *n*-6) content and limited antioxidant capacity, which promote faster oxidation. The moderate OIT for FX (22.34 min) corresponds to its high α-linolenic acid (C18:3 *n*-3) level (22.88%), which increases oxidation susceptibility despite its nutritional importance. This trend agrees with previous observations that triunsaturated fatty acids oxidize more rapidly due to a higher number of reactive double bonds [42].

Interestingly, total phenolic content (TPC) was not associated with OIT, since the residues with the highest TPC (SF and PS) showed relatively low induction times. This observation is consistent with general stability trends, which indicate that low oxidative stability may result from the dominant influence of unstable lipids, as the presence of polyunsaturated fatty acids (PUFA) in residual fat significantly lowers the Oxidation Induction Time (OIT). This effect is often more strongly negatively correlated with stability than the phenolic content itself; for instance, the -linolenic fatty acid (C18:3, a PUFA) showed a significant negative correlation with the Rancimat induction time (at 100 °C). Furthermore, in many stability analyses, it has been confirmed that a high concentration of free fatty acids (AV), which are formed as a result of hydrolysis and can accelerate oxidation, frequently overshadows the antioxidant activity of the total phenolic compounds [42,43,48].

### 3.3. Health-Related Lipid Indices (h/H, AI, TI)

The assessment of health-related lipid indices provided insight into the potential nutritional quality and cardiovascular implications of dietary fats. In the present study, three indices were calculated for the cold-press oil residues: the hypocholesterolemic/hypercholesterolemic ratio (h/H), the atherogenic index (AI), and the thrombogenic index (TI). These indices were first introduced by Ulbricht and Southgate [39] to describe the impact of individual fatty acids on plasma lipid metabolism. The h/H ratio reflected the balance between fatty acids that lowered serum cholesterol (hypocholesterolemic; mainly C18:1, C18:2, and C18:3) and those that raised it (hypercholesterolemic; primarily C14:0 and C16:0). The AI estimated the relationship between the sum of atherogenic fatty acids and the sum of anti-atherogenic ones, whereas the TI evaluated the thrombogenic potential of a given lipid profile based on its content of saturated, monounsaturated, and polyunsaturated fatty acids [45,49]. The obtained values of the indices were presented in Table 5. The h/H ratio ranged from 9.05 in blue poppy seed (PS) residue to 15.75 in walnut (WA) residue. The AI values varied between 0.064 and 0.111, and the TI values ranged between 0.099 and 0.256. These results indicated that all investigated residues possessed a favorable fatty acid composition, dominated by unsaturated fatty acids and characterized by a low content of atherogenic saturated fatty acids.

The highest h/H ratios were observed in the walnut and sunflower residues (15.75 and 14.74, respectively), reflecting their high proportions of linoleic (C18:2 *n*-6) and oleic (C18:1 *n*-9) acids. These fatty acids were known to exert hypocholesterolemic effects by modulating LDL/HDL cholesterol ratios and reducing cardiovascular risk [44]. Intermediate h/H values in the hemp (HS) and flaxseed (FX) residues (≈13) were attributed to their polyunsaturated fatty acid profiles, particularly the presence of α-linolenic acid (C18:3 *n*-3), while the lowest values in black cumin (BC) and blue poppy (PS) residues (<10) corresponded to their higher content of saturated fatty acids.

The low AI (0.064–0.111) and TI (0.099–0.256) values observed for all residues confirmed the low atherogenic and thrombogenic potential of these lipid fractions. Similar values were reported for cold-pressed flaxseed and hempseed oils, as well as for their respective press cakes, where AI and TI remained below 0.2 and 0.3, respectively [45]. Slightly higher indices recorded for sunflower and blue poppy residues were consistent with their elevated palmitic acid (C16:0) levels, the major SFA contributing to both indices.

From a nutritional standpoint, all analyzed residues exhibited lipid profiles markedly superior to those of animal fats, where AI typically exceeded 1.0 and TI 1.5 [49]. The combination of high h/H ratios and low AI and TI values demonstrated the favorable lipid quality of these cold-press by-products, supporting their potential utilization as ingredients in functional foods or nutraceutical formulations. Moreover, their composition—rich in unsaturated fatty acids and low in pro-atherogenic SFAs—also enhanced their suitability as substrates for microbial biotransformation within circular bioeconomy systems [41,43].

### 3.4. Valorization Potential of Cold-Press Residues for Microbial Transformation

Following the characterization of physicochemical properties and fatty acid profiles of the oilseed residues, a biological valorization strategy was undertaken to assess their suitability as substrates for microbial synthesis of lactones. Lactones constitute an important class of naturally occurring aroma compounds with high industrial relevance in the food, cosmetic, and pharmaceutical sectors [36]. Biotechnological approaches to lactone production have gained popularity in recent years due to their stereoselectivity, environmental friendliness, and regulatory acceptance as “natural” flavors within the European Union [30,50].

Several microbial pathways leading to lactone biosynthesis have been described in the literature, including degradation of hydroxylated fatty acids, reduction in unsaturated lactones [50], and α,ω-oxidation of alkanes and fatty acids [51]. One of the best-studied examples is the production of γ-decalactone from ricinoleic acid by *Y. lipolytica* [52,53].

It is worth noting that the aforementioned method of lactone synthesis from a naturally occurring hydroxy acid is associated with certain limitations. First, the yield of a given lactone depends entirely on the availability of a suitable precursor. Second, the industrial and commercial value of the precursors used is high and often competitive with the resulting lactone. Third, hydroxy fatty acids are neither abundant nor renewable, and obtaining ricinoleic acid (the lactone precursor) is associated with several health, seasonal, and economic issues related to the cultivation of the plant *Ricinus communis* from which it is derived. Therefore, there is a growing interest in other methods of lactone synthesis, based on readily available sources of unsaturated fatty acids [54,55].

The waste materials investigated in this study represent a rich source of lipids dominated by linoleic (C18:2) and oleic (C18:1) acids, which serve as precursors of δ-decalactone and γ-dodecalactone, respectively [56].

**Figure 1:**
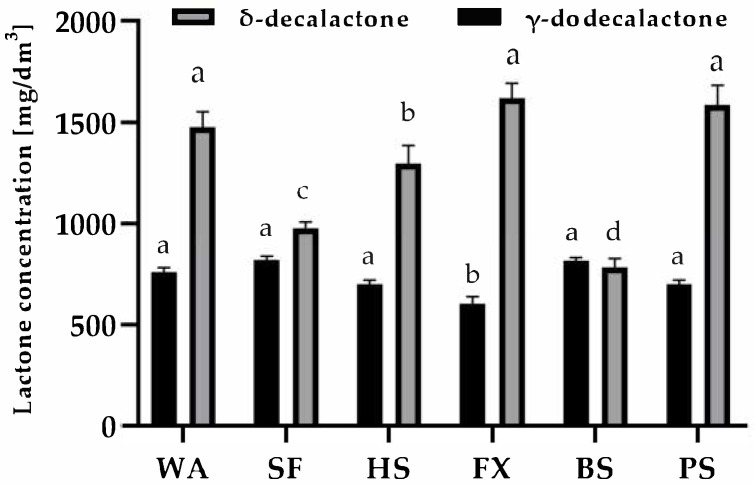
Concentrations of δ-decalactone and γ-dodecalactone in biotransformation mixtures after six days of incubation (GC-MS). Different letters indicate statistically significant differences (*p* < 0.05).

In the present work, a commercial lipase preparation, lactic acid bacteria (*L. plantarum*), and *Y. lipolytica* yeast were applied sequentially. Lipase-hydrolyzed triacylglycerols contained in the residues to release free fatty acids. *L. plantarum* facilitated the hydroxylation step, while *Y. lipolytica* catalyzed β-oxidation. Final lactone ring closure was achieved by lowering the pH to approximately 2. Volatile biotransformation products were identified and quantified using GC-MS after a six-day incubation period (Figure 1).

Regardless of the waste type applied, both γ-dodecalactone and δ-decalactone were detected in all biotransformation mixtures, confirming that the mixed cultures employed are capable of directly converting fatty acids present in waste materials into valuable aroma compounds [57]. In most substrates, markedly higher concentrations of δ-decalactone were observed—ranging from 1.48 to 1.62 g/dm^3^ for sunflower, blue poppy, walnut, and hemp residues—compared to 0.60–0.76 g/dm^3^ of γ-dodecalactone. This trend corresponds to the predominance of linoleic acid in the respective lipid fractions. In contrast, substrates with a higher proportion of oleic acid produced comparatively lower yields of γ-dodecalactone.

**Figure 2:**
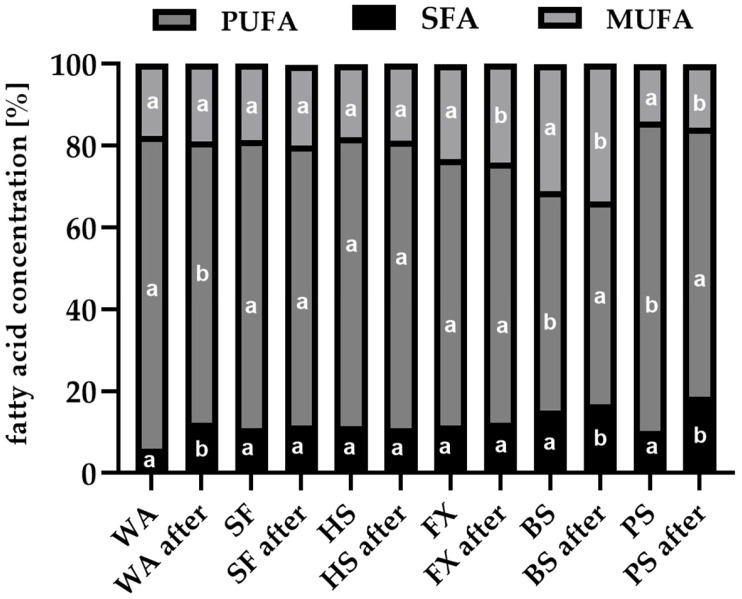
Relative changes in the share of saturated (SFA), monounsaturated (MUFA), and polyunsaturated fatty acids (PUFA) in residual oils before and after biotransformation. Different letters indicate statistically significant differences (*p* < 0.05).

Analysis of the post-reaction fatty acid profiles (Figure 2) indicated that polyunsaturated fatty acids (PUFAs) were metabolized more efficiently than monounsaturated fatty acids (MUFAs), highlighting their preferential involvement in biotransformation. Nevertheless, interpretation of these changes is limited by the percentage-based nature of the data and the possibility that fatty acids served both as biotransformation precursors and as carbon sources for yeast biomass growth. Similar observations regarding concurrent substrate metabolism and microbial proliferation have been reported by other authors [30,54].

The results of this study are promising and demonstrate the potential of unctuous lipid waste in the synthesis of lactones. The literature lacks data on the evaluation of this type of waste in the biosynthesis of fragrance compounds, making it difficult to comment on the reaction’s efficiency. Nevertheless, Małajowicz et al. [57] conducted research using raw rapeseed cakes in a two-pot biosynthesis process of γ-dodecalactone. Due to its high oleic acid content (over 62% of the lipid fraction), she used rapeseed cake in the biotransformation process. Thanks to the two-pot biotransformation, approximately 0.76 g of γ-dodecalactone/dm^3^ could be obtained using 100 g/dm^3^ of rapeseed cake.

The obtained results clearly demonstrate that cold-press residues can be biologically valorized without the need for prior purification of the lipid fraction. The applied microbial consortia efficiently catalyzed sequential steps of hydrolysis, hydration, β-oxidation, and lactonization, yielding industrially relevant quantities of lactones. Notably, the concentrations achieved here are relatively high despite the absence of process optimization, which is consistent with recent studies on oil-based substrates [54]. Among the examined residues, sunflower, blue poppy, and walnut wastes exhibited the highest potential for δ-decalactone synthesis. Future work should focus on optimization of process parameters—including aeration intensity, inoculum composition, pH control, and degree of lipid hydrolysis—to further improve conversion yields. Given their low cost, availability, and lipid richness, cold-press oilseed residues represent promising substrates for circular-economy-oriented biotechnological valorization

## 4. Conclusions

Cold-press oilseed residues were found to retain substantial amounts of lipids and unsaturated fatty acids, confirming their value as renewable biotechnological feedstocks. Physicochemical analyses and DSC revealed clear differences in oxidation stability, with hemp and black cumin residues being the most resistant and sunflower and walnut the least stable. All residues supported microbial biotransformation into γ- and δ-lactones, with the highest δ-decalactone concentrations achieved for sunflower, poppy, and walnut substrates.

The conversion of waste-derived lipids into value-added lactones represents a promising strategy for the sustainable utilization of industrial and food-processing residues. Nevertheless, several limitations must be acknowledged. The compositional variability of pasty lipid substrates, together with mass-transfer constraints inherent to heterogeneous lipid matrices, can negatively affect reaction efficiency and process reproducibility. These challenges become especially evident during scale-up, where consistent performance under fluctuating waste-stream characteristics is crucial.

Future research should focus on systematically optimizing this process to enhance its effectiveness. The application of advanced bioreactors equipped with real-time pH monitoring and automated control systems presents a promising approach to maintaining stable and efficient reaction conditions. Additionally, integrating well-designed feeding strategies—such as controlled batch or fed-batch modes—may enhance substrate availability while mitigating inhibitory effects. Equally important is the development of pretreatment methods that improve lipid accessibility, including emulsification and targeted enzymatic hydrolysis. The combination of these approaches has the potential to substantially increase efficiency and scalability of lactone production from waste lipids, supporting broader implementation.

## Figures and Tables

**Table 1:** List of oil pressing residues used as research material.

Code	Raw Material
WA	Walnut (*Juglans regia*)
SF	Sunflower (*Helianthus annuus*)
HS	Hemp seed (*Cannabis sativa*)
FX	Flaxseed (*Linum usitatissimum*)
BC	Black cumin (*Nigella sativa*)
PS	Blue poppy seed (*Papaver somniferum*)

**Table 2:** Physicochemical parameters of viscous residues derived from cold-pressed black cumin (BC), sunflower (SF), poppy seed (PS), hemp seed (HS), flax (FX), and walnut (WA) oils. Different superscript letters within the same column denote statistically significant differences (*p* < 0.05).

Code	Dry Mass (%)	Protein Content (%)	Fat Content (%)	Acid Value (mg KOH/g Oil)	Peroxide Value (meq O_2_/kg)	DPPH Inhibition (%)	Total Phenolic Content (mg GAE/g Oil)
WA	15.76 ± 2.24 ^c^	18.95 ± 0.00 ^a^	72.37± 3.34 ^a^	81.27 ± 1.36 ^a^	9.24 ± 1.30 ^e^	22.00 ± 0.01 ^c^	0.07 ± 0.00 ^b^
SF	5.50 ± 0.75 ^e^	16.32 ± 0.12 ^b^	76.17 ± 3.06 ^a^	57.35 ± 2.70 ^b^	26.33 ± 0.47 ^c^	15.00 ± 0.01 ^b^	0.11 ± 0.00 ^a^
HS	21.50 ± 1.75 ^b^	19.44 ± 0.00 ^a^	76.15 ± 1.63 ^a^	20.89 ± 1.66 ^c^	16.47 ± 2.63 ^d^	9.00 ± 0.01 ^b^	0.04 ± 0.00 ^c^
FX	25.00 ± 1.00 ^a^	15.58 ± 0.00 ^b^	70.10 ± 4.38 ^a^	75.60 ± 1.99 ^a^	52.16 ± 4.95 ^a^	16.00 ± 0.01 ^a^	0.09 ± 0.01 ^b^
BC	10.55 ± 2.55 ^d^	13.11 ± 0.00 ^c^	49.06.0 ± 4.16 ^b^	81.31± 2.33 ^a^	35.33 ± 4.63 ^b^	33.00 ± 0.02 ^d^	0.08 ± 0.00 ^b^
PS	21.89 ± 2.05 ^b^	17.31 ± 0.00 ^b^	75.46 ± 0.80 ^a^	77.74 ± 0.28 ^a^	8.76 ± 1.98 ^e^	13.00 ± 0.01 ^ab^	0.10 ± 0.00 ^a^

**Table 3:** Fatty acid profiles (%) of residues obtained from cold-pressed walnut (WA), sunflower (SF), hemp seed (HS), flax (FX), black cumin (BC), and poppy seed (PS) oils. Different superscript letters within the same row indicate statistically significant differences (*p* < 0.05). nd—not detected.

Symbol	WA	SF	HS	FX	BC	PS
C16:0	5.97 ± 0.08 ^c^	6.04 ± 0.28 ^b^	6.80± 0.26 ^b^	6.70 ± 0.44 ^b^	8.83 ± 0.41 ^a^	9.74 ± 0.01 ^a^
C18:0	nd	4.62 ± 0.08 ^a^	4.55 ± 0.23 ^a^	4.83 ± 0.23 ^a^	3.46 ± 0.00 ^b^	2.11 ± 0.08 ^b^
C18:1 *n*-9	17.58 ± 0.11 ^b^	18.54 ± 0.06 ^b^	17.96 ± 0.08 ^b^	20.95 ± 0.07 ^a^	21.42 ± 0.13 ^a^	14.08 ± 2.21 ^c^
C18:2 *n*-6	62.31 ± 0.23 ^c^	69.76 ± 0.31 ^b^	58.31 ± 0.21 ^d^	42.30 ± 0.18 ^e^	34.73 ± 0.99 ^f^	73.20 ± 2.28 ^a^
C18:3 *n*-3	14.15 ± 0 22 ^c^	nd	12.40 ± 0.16 ^c^	22.88 ± 0.11 ^a^	18.02 ± 0.64 ^b^	0.87 ± 0.01 ^d^
C20:0	nd	0.32 ± 0.07 ^b^	nd	nd	1.09 ± 0.01 ^a^	nd
C20:1 ^c^	nd	nd	nd	2.35 ± 0.08 ^b^	8.32 ± 0.23 ^a^	nd
C20:3 *n*-3	nd	nd	nd	nd	0.71 ± 0.12 ^b^	nd
C20:4 *n*-6	nd	0.73 ± 0.05 ^a^	nd	nd	nd	nd
C21:0	nd	nd	nd	nd	2.01 ± 0.03 ^a^	nd
C22:1 *n*-9	nd	nd	nd	nd	1.42 ± 0.01 ^a^	nd
SFA	5.97 ± 0.08 ^c^	10.98 ± 0.29 ^b^	11.34 ± 0.03 ^b^	11.53 ± 0.44 ^b^	15.39 ± 0.36 ^a^	11.85 ± 0.07 ^b^
MUFA	17.58 ± 0.11 ^b^	18.54 ± 0.06 ^b^	17.96 ± 0.08 ^b^	23.30 ± 0.07 ^b^	31.15 ± 0.11 ^a^	14.08 ± 2.21 ^b^
PUFA	76.45 ± 0.45 ^a^	70.49 ± 0.36 ^b^	70.70 ± 0.04 ^b^	65.17 ± 0.14 ^c^	53.46 ± 0.35 ^d^	75.67 ± 2.27 ^c^

**Table 4:** Oxidation induction time [min] of residues obtained from cold-pressed walnut (WA), sunflower (SF), hemp seed (HS), flax (FX), black cumin (BC), and poppy seed (PS) oils. Different superscript letters within the same row indicate statistically significant differences (*p* < 0.05).

Waste	Oxidation Induction Time [min]
WA	10.49 ± 3.61 ^c^
SF	3.12 ± 0.48 ^d^
HS	39.00 ± 2.87 ^a^
FX	22.34 ± 3.79 ^b^
BC	33.83 ± 2.82 ^a^
PS	12.15 ± 0.76 ^c^

**Table 5:** Hypocholesterolemic/hypercholesterolemic ratio (h/H), atherogenicity index (AI), and thrombogenicity index (TI) of viscous residues derived from cold-pressed walnut (WA), sunflower (SF), hemp seed (HS), flax (FX), black cumin (BC), and poppy seed (PS) oils. Different superscript letters within a column differ significantly (*p* < 0.05).

Waste	h/H	AI	TI
WA	15.75 ^a^	0.064 ^b^	0.114 ^c^
SF	14.74 ^a^	0.068 ^b^	0.239 ^a^
HS	13.04 ^b^	0.077 ^b^	0.150 ^b^
FX	13.21 ^b^	0.076 ^b^	0.113 ^c^
BC	9.58 ^c^	0.104 ^a^	0.099 ^c^
PS	9.05 ^c^	0.111 ^a^	0.256 ^a^

## Data Availability

The original contributions presented in this study are included in the article. Further inquiries can be directed to the corresponding authors.
